# Emergency departments reduce admissions

**DOI:** 10.1186/1757-7241-20-S2-P17

**Published:** 2012-04-16

**Authors:** Thomas Osterland, Dan Brun Petersen

**Affiliations:** 1 KU Planning, Region Zealand, Denmark; 2 Danish Society for Emergency Medicine, Denmark

## Background

It has been discussed whether emergency departments (EDs) reduce hospital admissions or not, and if the high turnover of patients results in higher readmission rates.

Region Zealand was one of the first regions in Denmark to establish EDs, where all the acute patients for the hospital should be received:

- Køge and Holbæk (no beds, doctors employed by and working only in the ED), Nykøbing Falster (16 beds, doctors on call from different specialties) and Slagelse (28 beds, doctors employed by and working only in the ED plus doctors from specialties dedicated to functions in the ED).

The departments have different organizational structures and the build up is not yet finished.

## Methods

The activity analysis was done on data from Sundhed counting all hospital discharges from specialty departments following acute admission. The study period was before (2009) and after (2011, the first 6 months) the establishment of the four EDs. The specialties involved were internal medicine, orthopaedic surgery and general surgery.

Furthermore the study included the overall readmission rate in the same period, defined by unspecified, acute readmissions within 30 days from discharge.

This analysis did NOT measure the activity in the emergency departments.

## Results

The numbers of discharges were reduced in all hospitals and all specialties, the lowest by 6% and the highest by 38.9%.

In general the greatest reduction was on internal medicine (Køge -6%, Holbæk -15% , Nykøbing -19%, Slagelse -33%). The reductions were even higher in 2010 in Køge (-13%) and Nykøbing (-33%) before two community hospitals in the proximity were closed.

The greatest reductions was at the two departments with short stay units / beds (Slagelse and Nykøbing), and most noticeable at Slagelse with doctors dedicated to the functions in the ED.

The rate of readmissions fell from 10.2% in 2008 to 8.4% in 2011. Compared to other regions, this was the highest change seen in the period.

## Conclusion

Establishing emergency departments in Region Zealand has reduced the number of acute admissions, especially in internal medicine. The reduction is most profound in department with short stay units and with designated doctors. The readmission rate was also reduced.

